# Molecular immunologic correlates of spontaneous latency in a rabbit model of pulmonary tuberculosis

**DOI:** 10.1186/1478-811X-11-16

**Published:** 2013-02-28

**Authors:** Selvakumar Subbian, Paul O’Brien, Nicole L Kushner, Guibin Yang, Liana Tsenova, Blas Peixoto, Nirmalya Bandyopadhyay, Joel S Bader, Petros C Karakousis, Dorothy Fallows, Gilla Kaplan

**Affiliations:** 1 Laboratory of Mycobacterial Immunity and Pathogenesis, The Public Health Research Institute (PHRI) Center at the University of Medicine and Dentistry of New Jersey (UMDNJ), 225 Warren Street, 07103, Newark, NJ, USA; 2 Biological Sciences Department, NYC College of Technology, Brooklyn, NY, USA; 3 Department of Biomedical Engineering and High-Throughput Biology Center, Johns Hopkins University, Baltimore, MD, USA; 4 Center for Tuberculosis Research, Department of Medicine, Johns Hopkins University School of Medicine and Department of International Health, Johns Hopkins Bloomberg School of Public Health, Baltimore, MD, USA

**Keywords:** *Mycobacterium tuberculosis*, Latent TB infection, Innate immunity, Th1 response, Genome-wide transcriptome, Host-pathogen interactions, Rabbit gene expression, Pathway analysis, Autophagy, Natural killer and dendritic cell activation

## Abstract

**Background:**

Infection of humans with *Mycobacterium tuberculosis* (Mtb) results in latent tuberculosis infection (LTBI) in 90-95% of immune competent individuals, with no symptoms of active disease. The World Health Organization estimates that 1.5 billion people have LTBI, which can reactivate in the setting of waning host immunity, posing a threat to global TB control. Various animal models have been used to study the pathogenesis of TB. However, besides nonhuman primates, rabbits are the only animal model that fully recapitulates the pathological features of human TB, including progressive disease with necrosis and cavitation or establishment of spontaneous latency.

**Results:**

We defined the molecular immunological correlates of LTBI establishment in a rabbit model of pulmonary infection with Mtb CDC1551. After aerosol infection, exponential bacterial growth was noted in the lungs for 4 weeks, followed by a significant decline by 12 weeks, resulting in the absence of cultivable bacilli by 24 weeks. We used rabbit whole genome microarrays to profile the lung transcriptome during the course of infection. At 2 weeks post-infection, gene networks involved in natural killer (NK) and dendritic cell (DC) activation and macrophage antimicrobial activities were highly upregulated. This was followed by upregulation of gene networks involved in macrophage and T cell activation and autophagy, peaking at 4 to 8 weeks. Concomitantly, host Th1, but not Th2 or inflammatory, immune response genes were significantly upregulated. Thus, the expression kinetics of genes involved in cross-talk between innate and adaptive immunity over the first 8 weeks post-infection were consistent with early efficient control of infection in the lungs. Interestingly, expression of many genes of the host innate and adaptive immune response pathways was downregulated at 12 weeks, suggesting that immune activation did not persist once bacilli began to clear from the infected lungs.

**Conclusions:**

Our results suggest that early activation of host innate immunity prior to efficient activation of T cell-mediated adaptive immunity but not inflammation is essential for establishment of LTBI in Mtb CDC1551-infected rabbits. We also show that T cell activation and the host adaptive immune response networks are dampened once bacterial growth is controlled, ultimately resulting in spontaneous LTBI.

## Lay abstract

Pulmonary infection by *Mycobacterium tuberculosis* (Mtb) results in latent tuberculosis infection (LTBI) in more than 90% of humans, with no symptoms of active disease. However, a weakening immune system can awaken LTBI, leading to active disease that can be transmitted to other persons. The host immune factors involved in establishing LTBI are poorly understood. In this study, we used a rabbit model of TB lung infection, which closely mirrors the characteristic tissue damage seen in humans, to identify molecular markers of host protective immune response during LTBI. Global gene expression analysis of Mtb CDC1551-infected rabbit lungs suggested that control of infection and establishment of LTBI was associated with activation of DC maturation, NK cell activation networks, and production of antibacterial molecules as early as 2 weeks of infection. At 4 and 8 weeks, genes that constitute the macrophage and T cell activation and autophagy networks, as well as Th1 type immune response genes, were upregulated. Activation of the majority of the host innate and adaptive immune response genes was dampened when the bacilli were cleared in the lungs. Taken together, our results suggest that early innate immunity, but not inflammation, prior to activation of adaptive immunity, are crucial for establishment of LTBI in rabbit lungs. Also, sustained activation of the host innate and adaptive immune response pathways is dampened once LTBI is established. These observation contrasts with the host response during active TB, where delayed onset and suboptimal activation of host immunity and extensive inflammation failed to control Mtb infection.

## Background

The World Health Organization (WHO) has estimated that nearly a third of the world’s population is infected by *Mycobacterium tuberculosis* (Mtb), the causative agent of tuberculosis (TB) [1]. While only 5-10% of Mtb infections cause primary active disease, this small proportion accounts for roughly 8.8 million new cases and 1.1 million deaths annually. In contrast, most Mtb-infected individuals do not develop active disease, but instead mount an immune response that effectively controls the primary infection, resulting in the establishment of latent TB infection (LTBI). Importantly, the infecting bacilli in LTBI are not necessarily killed but can persist in a viable, dormant state, which may be reactivated upon immune compromise of the host, even decades after initial infection, causing symptomatic disease (reactivation TB). LTBI is defined by a positive immune response to Mtb antigens in the absence of clinical signs or symptoms of active disease [2]. Because latently infected individuals constitute a large reservoir of potential TB cases and new sources of infection, LTBI poses a significant problem for global TB control [3].

In humans, containment of bacillary growth and prevention of dissemination of Mtb from the primary infection site in the lungs to other tissues is achieved by the formation of granulomas [4]. These are well organized cellular structures, comprised mostly of mononuclear leukocytes and granulocytes recruited from the circulation to the site of infection, where they accumulate around Mtb-containing macrophages [5,6]. Available experimental data suggest that the magnitude and kinetics of leukocyte recruitment and activation of the immune cells within the granulomas, as well as the phenotype of the infecting bacilli, all contribute to determining whether the outcome of Mtb infection will be LTBI or active disease. However, how the components of the host immune response to Mtb infection are regulated is not fully understood [7,8]. To address these questions, several animal models of TB have been developed, including mice, guinea pigs, rats, rabbits, and nonhuman primates (NHP) [9,10]. However, with the exception of rabbits and NHP, no other animal models faithfully recapitulate the spectrum of clinicopathological features found in Mtb-infected humans, from progressive (primary) TB to LTBI and reactivation (secondary) TB [10,11].

In the rabbit, to a greater extent than other animal models of TB, the nature of the infecting Mtb strain significantly influences host-pathogen interactions and determines the outcome of infection [12,13]. The clinical Mtb strain CDC1551 has been shown by us and others to be highly immunogenic but not hypervirulent in animals [14]. We have shown that aerosol infection of rabbits with Mtb CDC1551 results in an early phase of transient limited bacillary growth, followed by spontaneous clearance of the infection, demonstrated by an absence of detectable colony forming units (CFU) in the lungs, liver and spleen [10,15]. As in humans, LTBI in CDC1551-infected rabbits can be reactivated upon treatment with immunosuppressive agents. Control of CDC1551 infection in rabbits is associated with small, well-differentiated lung granulomas. Concurrent with bacillary clearance, the granulomatous lesions resorb with time and the lungs regain a normal appearance [15,16]. Thus, we have established a rabbit model of LTBI that mimics the clinical responses to Mtb infection seen in the majority of immune-competent humans.

We have previously shown that progressive TB in rabbits infected with Mtb HN878 is associated with a slow and suboptimal activation of host innate and adaptive immune cells and extensive activation of the host chronic inflammatory response. Moreover, the sustained presence of activated CD4+ and CD8+ T cells throughout the course of infection is driven by the bacillary load in the lungs [17]. In contrast, in CDC1551-infected rabbits, an early and robust activation of the host leukocyte response is noted. In these animals, activation of macrophages and CD4 T cells peaks by 4 weeks and gradually declines by 12 weeks post-infection, in association with resolution of the pathology and clearance of bacilli from the lungs [15]. In the present report, we have used a transcriptomic approach to define the molecular determinants of the host immune response associated with establishment of LTBI in the lungs of CDC1551-infected rabbits.

## Results

### Rabbit infection and qualitative analysis of microarray gene expression data

**Figure 1:**
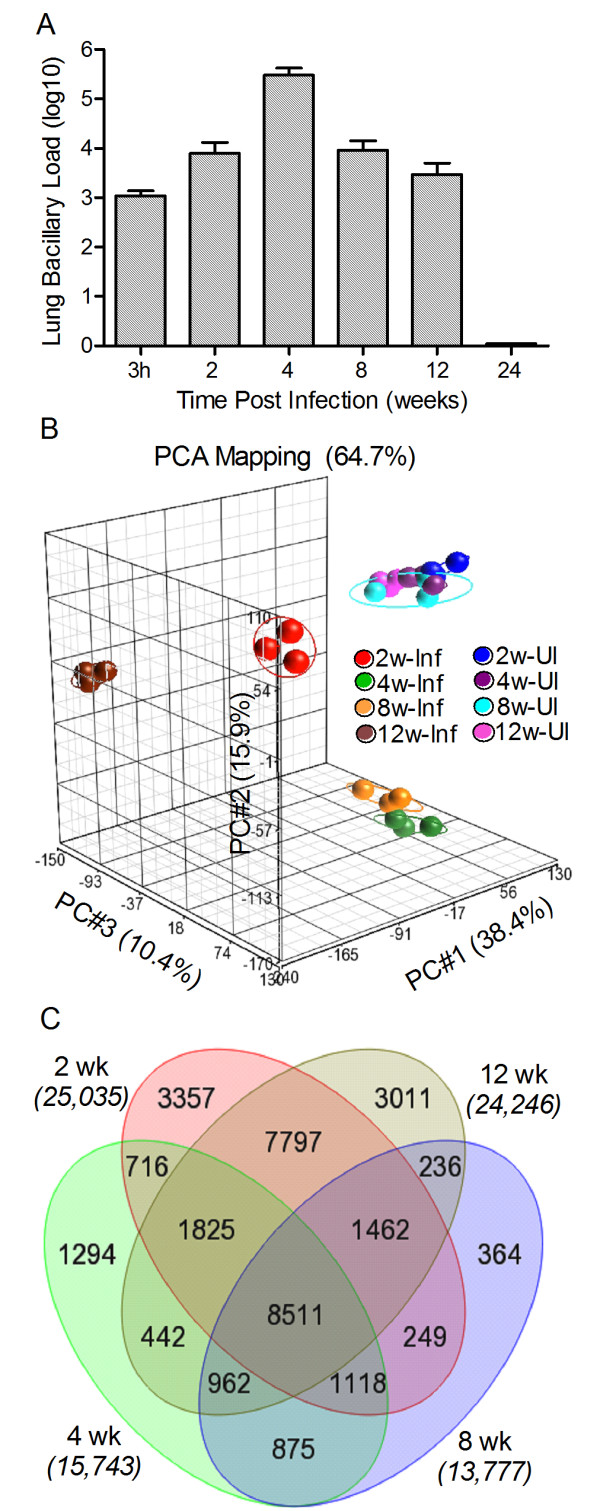
**Bacillary growth and global transcriptome analysis of rabbit lungs during Mtb CDC1551 infection.** Bacterial CFU in the lungs were enumerated up to 24 weeks and microarray analysis of gene expression profile was performed in the lungs of Mtb-infected rabbits at 2, 4, 8 and 12 weeks post-infection and compared to the uninfected animals. **A**: Total lung bacillary load determined by plating organ homogenates from each infected rabbit on agar plates. For each animal, about 30% of the entire lung, comprising all 5 lung lobes, was used to prepare the homogenates. **B**: PCA mapping of data points obtained from pooled samples from uninfected (n = 3) or one experiment from each of the three biological replicates from Mtb-infected rabbit lungs at each time point (n = 3 per time point). Eclipse drawn around the triplicate data points for each time point represents area spanning two-fold standard deviation from the median expression levels. **C**: Venn diagram showing the number of statistically significantly differentially expressed genes (SDEG) by Mtb infection of rabbit lungs at 2, 4, 8 and 12 weeks post-infection. The genes were selected based on a False Discovery Rate (FDR) of 5% (equivalent to *p* ≤0.05).

We have previously described the establishment of LTBI in a rabbit model of pulmonary TB, using the clinical Mtb isolate CDC1551 to infect animals via the respiratory route [15]. In the present study, rabbits were infected with 1.08 ± 0.31 ×10^3^ colony forming units (CFU) of CDC1551 implanted into the lungs. Over the course of infection, the lung bacillary load increased to 7.9 ± 5.3 ×10^3^ (2 weeks) and 3.0 ± 1.3 ×10^5^ (4 weeks), then decreased to 9.24 ± 4.9 ×10^3^ (8 weeks) and 2.97 ± 2.07 ×10^3^ (12 weeks), finally becoming undetectable (detection limit <25 CFU) by 24 weeks post-infection (Figure 1A). To define the kinetics of immunological changes occurring in the lungs in response to CDC1551 infection, whole genome expression analysis was carried out using the Agilent rabbit microarray, which contains 43,803 probes, each 60 nucleotides long, derived from RefSeq (Release 29), UniGene (Build 11) and Ensembl (Release 49). As a reference for the microarray analysis, we pooled total RNA from the lungs of 3 uninfected, naïve animals and prepared cDNA. We then prepared cDNA from each individual test rabbit (n = 3 per time point) following 2, 4, 8 and 12 weeks of infection. Changes in the lung transcriptome of each infected rabbit were expressed as a ratio/fold change of gene expression relative to the reference (pooled uninfected animals). The quality of the microarray data was assessed by Principal Component Analysis (PCA) (Figure 1B). The three dimensional PCA plot showed a 38.4%, 15.9%, and 10.4% variation in gene expression across different biological replicates, at different time points and between each infected animal and the uninfected reference in the X, Y and Z axis, respectively. PCA analysis revealed that i) data from the uninfected (pooled) reference clustered together; ii) data from biological replicates of the CDC1551-infected samples at each of the time points were tightly clustered, indicating a good reproducibility of variations in the X, Y and Z axis (64.7%) among biological replicates; iii) the cumulative data at 2, 4, 8 and 12 weeks post-infection segregated far from each other and farther from the uninfected reference, suggesting dynamic shifts in the infected lung transcriptome over time and a significant separation between the CDC1551-infected and uninfected groups (Figure 1B).

### Global changes in the rabbit lung transcriptome induced by Mtb CDC1551 infection

To define global changes in the Mtb-infected rabbit lung transcriptome relative to uninfected animals, expression values of the 43,603 probes in the microarray at all time points were analyzed by 2-way ANOVA, after adjusting the raw data for background signals. Significantly differentially expressed genes (SDEG) were selected based on a false discovery rate of 5% between the pooled uninfected and individual Mtb-infected samples at each time point. The total number of SDEG peaked at 2 weeks (25,035 genes), followed by a reduction in these numbers at 4 (15,743 genes) and 8 (13,777 genes) weeks of Mtb infection. By 12 weeks post-infection, the number of SDEG had increased to 24,246 (Figure 1C). Among the SDEG, the percent of upregulated genes increased gradually from 2 (47.4%) to 4 (47.9%) and 8 (48.8%) weeks, followed by a reduction at 12 weeks (44.6%) post-infection, suggesting temporal changes in the nature of the rabbit host response during CDC1551 infection. A common subset of 8511 genes was found among the total SDEG at all time points tested.

### Functional analysis of differentially expressed genes

We next analyzed the annotated SDEG to evaluate functional contributions to the host response to Mtb infection using Ingenuity Pathway Analysis (IPA), as described [17]. Human, mouse, and rat ortholog of rabbit genes from the IPA knowledgebase were used for the functional pathway/network analysis. Among the SDEG, 16,884 (2 weeks), 10,412 (4 weeks), 11,125 (8 weeks) and 16,381 (12 weeks) genes were eligible for pathway/network analysis by IPA. Comparative gene ontology analysis revealed that the cell death, cellular growth and proliferation, cellular assembly, cell movement, cell to cell signaling, and molecular transport pathways were among the top cellular functions affected by CDC1551 infection (Additional file 1: Table S1). Importantly, with the exception of cell-to-cell signaling and interaction, the number of SDEGs representing these functions peaked at 2 weeks and declined gradually until 8 weeks, followed by a moderate increase at 12 weeks post-infection. The number of SDEGs involved in cell-to-cell signaling and interaction increased more than four-fold from 2 to 4 weeks (170 vs. 682) and then declined gradually at 8 (>2 fold) and 12 weeks post-infection (Additional file 1: Table S1).

We dissected the gene ontology data to determine the top canonical pathways involved in these cellular functions (Additional file 2: Table S2). At 2 weeks, the most significantly affected pathways included signaling through integrin linked kinase (ILK), T cell receptor, IL-1 and CCR3, as well as production of nitric oxide and reactive oxygen species. In contrast, SDEGs of the leukocyte extravasation signaling, Fc-gamma mediated phagocytosis in macrophages and monocyte, and IL-8 signaling pathways peaked at 4 weeks. The IL-6, mTOR, CXCR4 and PI3K signaling pathways were the most significantly affected at 12 weeks post-infection (Additional file 2: Table S2). Overall, the gene ontology and canonical pathway analysis suggests a significant and dynamic impact on cellular pathways that are important for host immunity during CDC1551 infection. These findings were further corroborated by our extended analysis of transcriptional regulators among the SDEG using z-scores, which infer the activation status of a pathway based on the expression pattern of the relevant transcription factors and molecules included in the IPA pathway (Additional file 3: Tables S3 and Additional file 4: Table S4). A positive z-score implies potential activation and a negative z-score indicates potential inhibition of the pathway (Ingenuity® Systems, Inc. Redwood City, CA).

### Validation of microarray by qPCR

To validate the microarray data, we measured transcript levels of selected genes by qPCR in the 3 biological replicates of Mtb-infected rabbit lung samples at each time point and compared the results to levels in the pooled reference from uninfected animals. Seventeen genes encoding cytokines/chemokines (*MIP1*, *MCP2*, *IFNG*, *IL18*, *IL2*, *IL4* and *CTLA4*), cell surface receptors (*MSR1*, *CD28*, *IL1R*, *CD80* and *CD86*), and host defense and regulatory molecules (*CRP*, *CAP18*, *LOX1*, *NOS2* and *IRF1*) representative of networks known to be associated with the host immune response were selected for qPCR analysis. As shown in Additional file 5: Table S5, the temporal patterns and expression levels of the selected genes were generally consistent between the microarray and qPCR experiments, thus validating the rabbit microarray gene expression data.

### Selected network analysis of differentially expressed genes

Based on the functional analysis of the SDEG affected by CDC1551 infection, we selected six networks known to play crucial roles in the host cellular immune response, including natural killer (NK) cell activation, dendritic cell (DC) maturation, immune cell antibacterial activity, activation of macrophages and T cells and autophagy. These networks were evaluated for gene expression over the duration of the experiment.

#### Natural killer (NK) cell activation network

**Figure 2:**
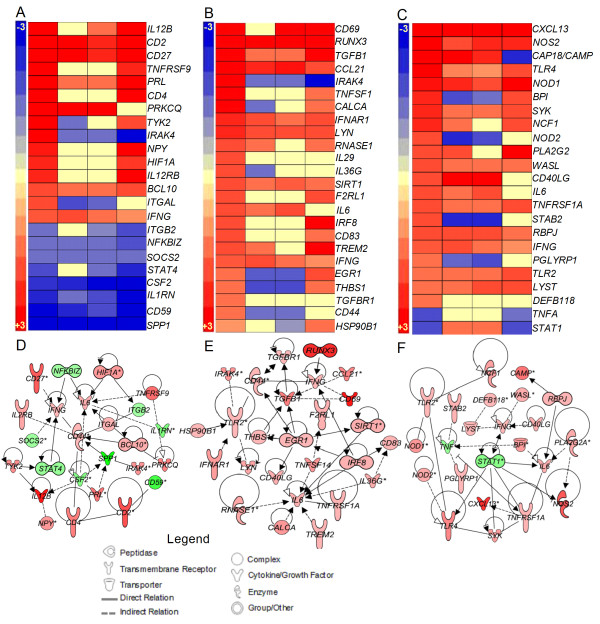
**Gene expression analysis of the NK cell activation, DC maturation and host cell antimicrobial defense networks.** Intensity plot and network analysis was derived from the SDEG altered by Mtb infection compared to the uninfected reference at 2, 4, 8 and 12 weeks post-infection using Partek Genomics Suite (PGS) and Ingenuity Pathway Analysis (IPA) software. Intensity plot (**A**) and interaction map (**D**) of NK cell activation network. Intensity plot (**B**) and interaction map (**E**) of DC maturation network. Intensity plot (**C**) and interaction map (**F**) of host cell antimicrobial defense network. The scale for all intensity plots ranges from +4 (red) to -4 (blue). Red symbols in the networks indicate up-regulation; green denotes down-regulation of gene expression. For D, E and F, the gradation in the color intensity of gene symbols is proportional to their relative expression level at 2 weeks post-infection.

NK cells are a subset of lymphoid cells that contribute to the host innate immune response against pathogens by producing Th1 cytokines required for the early activation of DCs and macrophages [18,19]. The expression pattern of genes involved in the activation of NK cells in Mtb-infected rabbit lungs was evaluated over time (Figure 2A and D). Twenty-four genes in this network were significantly differentially expressed, including those encoding cytokines and chemokines (*IL12B*, *IL1RN*, *PRL*, *IFNG, IL6 SPP1* and *CSF2*), leukocyte transmembrane receptors (*IL2RB, CD27*, *CD4* and *CD2*), enzymes (*CD44*, *TYK2*, *IRAK4, MALT1* and *PRKCQ*), transcriptional regulators (*NFKBIZ*, *HIF1A*, *STAT4* and *BCL10*) and other cytoplasmic (*SOCS2*) and plasma membrane molecules (*CD59, CD244, ITGB2 and ITGAL*). Of these genes, 14 were upregulated as early as 2 weeks post-infection. The number of upregulated genes decreased to 5 and 6, and increased to 12, at 4, 8 and 12 weeks post-infection, respectively. All genes that were downregulated at 2 weeks (9 genes) remained either downregulated or not significantly expressed at 4, 8 and 12 weeks (Figure 2A). This early activation of NK cells suggests a central role for these cells in initiating the host immune response to CDC1551 infection.

#### Dendritic cell (DC) maturation network

Antigen-presenting DCs engulf the infecting bacilli and help to sustain an anti-microbial response [20]. In addition to antigen presentation, DCs also produce cytokines that contribute to the activation of cells of the host innate and adaptive immune response, including NK and T cells. The DC maturation network genes encode cytokines, chemokines and their receptors (*IL6*, *TGFB1, IFNG, CCL19, CCL21, IL29, IL36G, TNFSF14* and *CD40LG)*, cell surface receptors that aid in recognition and internalization of microbes (*CD83*, *IFNAR1, TREM2, TNFRSF1A, TLR2* and *F2RL1)*, transcriptional regulators (*IRF8*, *EGR1*, *SIRT1* and *RUNX3*) and enzymes (*LYN*, *CD44*, *IRAK4*, *THBS1*, *CALCA, RNASE1* and *TGFBR1*) (Figure 2B and E). All 24 SDEGs in the DC maturation network were over expressed at 2 weeks of infection. The number of induced genes declined to 10 and 8 at 4 and 8 weeks, before increasing to 18 at 12 weeks post-infection, respectively (Figure 2B). Seven of the genes, including *IFNG, RUNX3, TGFB1, CCL21, IFNAR1, LYN* and *SIRT1*, were upregulated at all time points tested. Taken together, these results suggest an early activation of DC functions, indicative of an early capacity to present antigens and activate T cell-mediated adaptive immunity upon Mtb infection.

#### Antimicrobial defense network

Activated immune cells, such as macrophages and DCs eradicate the engulfed pathogens via production of reactive oxygen and nitrogen species and by other microbicidal molecules, such as defensin and cathelicidin [21]. To determine the contributions of these functions to effective control of CDC1551 in the infected rabbit lungs, we analyzed the expression levels of genes associated with the antimicrobial activities of phagocytes (Figure 2C and F). The list of SDEGs in this network included those encoding cell membrane receptors (*TLR2*, *TLR4*, *PGLYRP1*, *TNFRSF1A* and *STAB2*), cytokines and chemokines (*IL6*, *IFNG*, *CXCL13*, *TNFA* and *CD40LG*), enzymes (*NOS2, PLA2G2A, SYK, MMP7 and NCF1*), transcriptional regulators (*RBPJ, STAT1* and *LYST*) and other cytoplasmic molecules (*BPI, NOD1, NOD2, WASL, CAMP* and *DEFB118*). Of the 24 genes in this network, 21 were up-regulated as early as 2 weeks and most of these remained upregulated at 4 (17 genes), 8 (15 genes) and 12 weeks (14 genes) post-infection. An unexpected observation was the downregulation of *TNFA* levels throughout the 12 weeks of infection. Thus, the pattern of expression of host cell antimicrobial defense network genes is consistent with the observed control of lung bacillary load in the absence of chronic inflammation in the CDC1551-infected rabbit lungs (Figures 1 and 2).

#### Macrophage activation network

**Figure 3:**
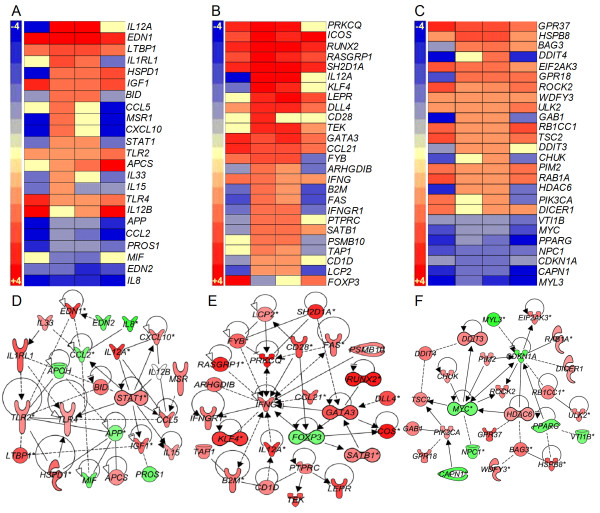
**Gene expression analysis of macrophage and T cell activation and autophagy networks.** Intensity plot and gene interaction network analysis was derived from the SDEG at 2, 4, 8 and 12 weeks post-infection using PGS and IPA software. Intensity plot (**A**) and interaction map (**D**) of macrophage activation network. Intensity plot (**B**) and interaction map (**E**) of T-cell activation network. Intensity plot (**C**) and interaction map (**F**) of host cell autophagy induction network. The scales for intensity plots range from +3 (red) to -3 (blue) (A) or +5 to -5 (B) or +4 to -4 (C). Red symbols in networks indicate up-regulation; green denotes down-regulation of gene expression. For D, E and F, the gradation in the color intensity of gene symbols is proportional to their relative expression level at 4 weeks post-infection. The legend for D, E, and F is same as in Figure 2.

Alveolar macrophages and blood-derived mononuclear phagocytes recruited to the site of infection are the central immune cells involved in the formation of granulomas, a structure crucial for containment of bacterial dissemination [6,7]. There are 23 genes in the macrophage activation network that encode cell surface receptors (*IL1RL1, TLR2, TLR4* and *MSR1*), cytokines and chemokines (*IL33, CCL2, CXCL10, IL15, CCL5, IGF1, MIF*, *EDN1* and *EDN2*), enzymes (*HSPD1* and *PTGS2*), a transcriptional regulator (*STAT1*), and other extracellular molecules (*PROS1, APP, LTBP1, APCS* and *APOH*) (Figure 3A and D). While only 5 out of the 23 genes were upregulated at 2 weeks, both the number of upregulated genes and their expression levels peaked at 4 weeks (15 genes), and then gradually decreased at 8 (10 genes) and 12 weeks (7 genes) post-infection. In contrast, the number of downregulated genes declined from 2 weeks (14 genes) to 4 (8 genes), and 8 weeks (7 genes), before peaking at 12 weeks (15 genes). Importantly, inflammatory cytokine genes, such as *IL15*, *IL33* and *IL8,* were either downregulated at all the time points tested or upregulated only transiently at 4 weeks (Figure 3A). Consistent with the kinetics of expression of the molecular markers of macrophage activation, a significant percentage of CD14^+^ cells produced TNF-α in the lungs and spleen (10.5% and 9.1% respectively) of CDC1551 infected rabbits at 4 weeks post infection [15]. The proportion of these cells increased at 8 weeks (38.4% and 15% respectively) and then declined (7.8% and 6.8% respectively) by 12 weeks. Moreover, expression of genes for matrix metalloproteases, that are involved in tissue destruction and remodeling (*MMP9*, *MMP12* and *MMP14*) and are regulated by chronic inflammation, were downregulated, while the negative regulator (*TIMP1*) of these proteases was upregulated in the lungs of CDC1551-infected rabbits at all time points [15]. Consequently, immunohistologic identification of collagen in the granulomas, as revealed by the trichrome staining of infected lung sections, was very low or nonexistent in the CDC1551-infected rabbits at 12 weeks [15].

#### T cell activation network

A number of subpopulations of T cells (CD4, CD8 and Tregs) play a central role in the adaptive immune response against Mtb infection [22]. We have previously reported that T cell activation in response to Mtb CDC1551 infection of rabbits was induced early, peaked at 4 to 8 weeks and declined as the bacillary load declined [15]. To understand the activation status of T cells during Mtb infection of rabbit lungs, we evaluated the transcription pattern of genes that constitute the T cell activation network (Figure 3B and E). The SDEG in the IFN-γ mediated, T-cell activation network are involved in transcriptional regulation (*KLF4, GATA3, FOXP3, SATB1* and *RUNX2*), cellular signaling (*LEPR, B2M, CD1D, IFNGR1*, and *FAS*), cytokines and chemokines (*IL12A, IL5, CCL21, IFNG* and *CXCL10*), enzymes (*PTPRC, TEK, BLM, PSMB10* and *PRKCQ*) and other cytoplasmic/nuclear molecules (*TAP1, RASGRP1, SH2D1A, LCP2, FYB, ARHGDIB, ICOS* and *DLL4*). Of these 28 genes, 11 were upregulated and 13 were downregulated at 2 weeks. With the exception of FOXP3, all genes in the T-cell activation network showed greatest expression at 4 weeks, and 24 out of 27 genes were also upregulated at 8 weeks. Thereafter, the number of upregulated genes declined to 13 (12 weeks), while the number of downregulated genes declined (10 genes). Importantly, upregulation of *FOXP3*, the negative regulator of T cell proliferation is consistent with the expression pattern of other activating genes in this network. Thus, the pattern of gene expression in the T cell activation network suggests that this pathway was activated starting at 2 weeks, peaking at 4 weeks, followed by a slow decline to 12 weeks post-infection. Similar to these kinetics, the peak proliferative capacity of CD4^+^ spleen T cells was observed at 4 weeks (67%) post-CDC1551-infection that declined to below 20% at 8 and 12 weeks. Furthermore, about 50% of the CD8^+^ spleen T cells were proliferating at 4 weeks. These numbers increased moderately to peak at 8 weeks (57%) followed by a decline at 12 weeks (55%). Importantly, proliferation of both the CD4^+^ and CD8^+^ spleen T cells dropped to about 10% at later time points (20 and 24 weeks post-infection) [15].

#### Autophagy induction network

Autophagy is a highly conserved process that helps in removing cellular debris emerging from normal homeostasis and from damage due to infection and inflammation [23]. Autophagy has been implicated in the elimination of Mtb by IFN-γ activated macrophages and has been shown to endow the autophagosomes of immune cells with antimicrobial activities [24,25]. Therefore, we studied the network that induces autophagy in immune cells (Figure 3C and F). Of the 26 genes in this network, 12 were upregulated and 14 were downregulated at 2 weeks. The number of upregulated genes increased to 15 and 19 at 4 and 8 weeks, respectively. Over the same period, only 7 genes were downregulated. However, the number of upregulated genes declined (13 genes) and downregulated genes increased (12 genes) at 12 weeks post-infection. Overall, the pattern of gene expression in this network suggests a gradual induction of autophagy from 2 weeks, peaking at 4 and 8 weeks, before moderately declining at 12 weeks, in parallel with the containment of bacillary growth in the infected rabbit lungs.

### Canonical pathway analysis

In this study, we observed an early activation of molecular networks of the innate (NK cell activation, DC maturation and antimicrobial activities) immune response manifest at 2 weeks of infection. In contrast, networks of macrophage activation and the acquired immune response (T cell activation) and autophagy peaked later, at 4 and/or 8 weeks post-infection. To better understand the interactions between these two arms of host immunity, we studied two canonical pathways: 1) communication between DC and NK cells at 2 weeks and 2) interaction between innate and adaptive immune cells at 4 weeks post-infection. These canonical pathways were constructed by IPA using experimentally confirmed gene expression data from the literature.

The interaction between various soluble and surface molecules that are differentially expressed in DC and NK cells plays a crucial role in the activation of both cell types and in the induction of a T cell-mediated adaptive immune response. In the DC and NK cell communication pathway, expression of *TNFR2, LTBR, LTα1β2*, *TREM2, CD83, LFA, IL15R* and *IL6* was upregulated, while that of *FAS*, *Nectin2*, *HLA-DR*, *GM-CSF and IL18* was downregulated in DC. Similarly, *IFNG*, *LTα1β2*, *CD69*, *CD40L, TNFR2, IL15R, LFA-1* were upregulated and *FAS, TNFA, TRAIL* and *GM-CSF* were downregulated in NK cells (Additional file 6: Figure S1). The gene expression pattern suggests an active interaction between DC and NK cells early (2 weeks) during infection. For example, upregulation of IFN-γ and CD69 by NK cells could activate DCs to increase production of IL-12 and IL-6. Similarly, engagement of TLR on DCs by Mtb surface molecules could induce the expression of IL-12 by DCs, which in turn could activate NK cells to express the genes encoding IFN-γ through upregulation of *CD69*. In summary, the canonical pathway for the communication between DC and NK cells is consistent with and supported by our observations on individual networks involved in the activation of these two immune cell types, which are highly upregulated as early as 2 weeks post-infection.

In addition to a strong innate immune responses, successful containment of TB infection also requires an effective adaptive immune response [8]. Though activation of the adaptive immune response was initiated at 2 weeks, maximal expression of the majority of genes in these networks was not observed until 4 or 8 weeks. We therefore studied the interaction between the innate and adaptive immune response using the gene expression data from 4 weeks post-infection (Additional file 7: Figure S2). CD4 T, CD8 T, and B cells constitute the central effector cells of the adaptive immune response. Macrophages, NK cells, and DCs, all cells of the innate immune response, provide antigen presentation and a co-stimulatory function in lymphocyte activation. Significantly upregulated expression was noted for the innate immunity markers *TLR*, *IL12*, *HLA1, CD40L*, *IP-10*, *IFNβ*, *RANTES,* and *IL15* and the adaptive immunity markers *CD28*, *IFNG, IL5, BCMA,* and *IL4*. In contrast, expression of innate and adaptive immune response genes *GMCSF*, *IL1*, *IL8* and *BLyS* was downregulated. The expression pattern of genes involved in the interaction between the innate and adaptive immune response suggests activation of both CD4 and CD8 T cells by innate immune cells. These observations are consistent with the results from the individual networks involved in activation of T cells, and IFN-γ mediated cellular functions.

### Expression of Th1/Th2 immune response genes in Mtb-infected rabbit lungs

**Figure 4:**
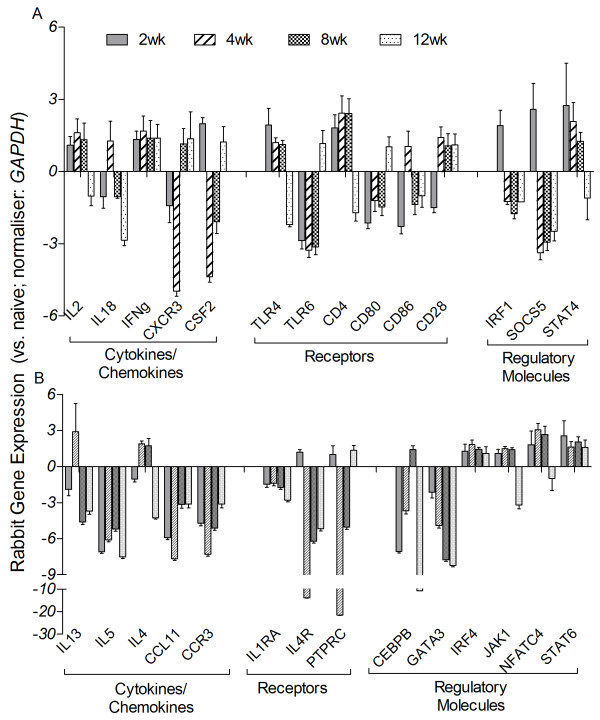
**qPCR analysis of rabbit genes involved in the Th1 and Th2 type immune response.** cDNA was synthesized from the total lung RNA from pooled, uninfected (n = 3) and three independent biological replicates of Mtb-infected rabbits at each time point (2, 4, 8 and 12 weeks post-infection) and used in the qPCR experiments. Genes involved in Th1 (**A**) and Th2 (**B**) type immune response were selected from published literature. Gene-specific oligonucleotide primers were used to amplify the test genes. Levels of *GAPDH* in each sample were used to normalize the expression of test genes. Relative gene expression was calculated from the expression levels of each test gene in the uninfected (naïve) and Mtb-infected samples after normalization against *GAPDH* levels. The experiments were repeated at least three times with lung total RNA from three biological replicates. Values plotted are median ± standard error.

Next we studied the transcript level of genes involved in establishing the Th1 and Th2 type immune response in Mtb-infected rabbit lungs at 2, 4, 8 and 12 weeks post-infection by qPCR analysis (Figure 4). Among the selected list of genes that encode cytokines/chemokines, *IL2*, *IL18*, *IFNG*, *CXCR3* and *CSF2* are involved in the Th1 pathway, while *IL13*, *IL5*, *IL4*, *CCL11* and *CCR3* are associated with the Th2 pathway. Receptors (*TLR4*, *TLR6*, *CD4*, *CD80*, *CD86* and *CD28*) and regulatory molecules (*IRF1*, *SOCS5* and *STAT4*) of the Th1 as well as Th2 pathway (*IL1RA*, *IL4R* and *PTPRC*; receptors, *CEBPB*, *GATA3*, *IRF4*, *JAK1*, *NFATC4* and *STAT6*; regulatory molecules) were also evaluated. Of the 14 Th1 response genes tested, the expression levels of 8 genes (*IL2*, *IFNG*, *CSF2*, *TLR4*, *CD4*, *IRF1*, *SOCS5* and *STAT4*) were significantly elevated at 2 weeks and several of these (*IL2*, *IFNG*, *TLR4*, *CD4* and *STAT4*) had similar expression levels at 4 and 8 weeks post-infection (Figure 4A). While expression of *IFNG* was upregulated at all time points, *IRF1* and *SOCS5* (2 weeks), *IL18* and *CD86* (4 weeks), and *TLR6* and *CD80* (12 weeks) were only transiently upregulated. *CD28* expression was upregulated from 4 to 12 weeks and *CXCR3* expression was upregulated at 8 and 12 weeks post-infection. Notably, none of these Th1-type response genes were downregulated, compared to levels in uninfected rabbits, at any of the time points tested. The general pattern of expression of the Th1 type response genes suggests an early activation (2 weeks) of this pathway that was sustained up to 8 weeks post-infection.

Contrary to the pattern of Th1-type response gene expression, 5 of the 14 Th2 type response genes (*IL5*, *CCL11*, *CCR3*, *IL1RA* and *GATA3*) were significantly downregulated at 2, 4, 8 and 12 weeks post-infection (Figure 4B). In addition, except for 4 of the regulatory genes (*IRF4*, *JAK1*, *NFATC4* and *STAT6*), many of the other genes in this pathway were only transiently expressed at 2 (*IL4R* and *PTPRC*) or 4 and/or 8 (*IL13*, *IL4* and *CEBPB*) weeks post-infection. While *IRF4* and *STAT6* had similarly elevated expressions at 2, 4, 8 and 12 weeks, *JAK1* and *NFATC4* were upregulated at comparable levels from 2 to 8 weeks and then downregulated at 12 weeks post-infection. In summary, the expression pattern of genes in the Th2-type response suggests sustained inhibition of this pathway, starting as early as 2 weeks and maintained throughout the experiment.

## Discussion

**Figure 5:**
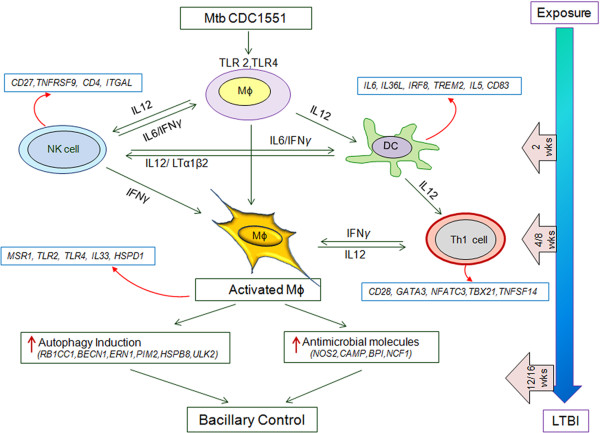
**Schematic representation of control of Mtb CDC1551-infection and establishment of LTBI in rabbit lungs.** SDEG in the rabbit lungs at 2, 4, 8 and 12 weeks post-infection was used for modeling. The blue arrow (right) indicates the kinetics of the host protective immune response to infection in the rabbit lungs over time, from exposure to establishment of LTBI. The gene symbols in the boxes next to immune cells were obtained from SDEG in CDC1551-infected rabbit lungs at each time point.

Based on our genome-wide transcriptional and cellular function network analysis, we propose a model for the control of infection and establishment of latency in the lungs of Mtb CDC1551-infected rabbits (Figure 5). Upon engagement of Mtb through TLRs, macrophages and DCs produce IL-12, which activates cells of the innate response (NK cells and DCs) and initiates the activation of adaptive immunity (T cells) as early as 2 weeks. By 4 weeks, Th1 cells are fully activated and, together with activated NK cells, produce IFN-γ, thereby activating macrophages to produce antimicrobial molecules and induce autophagy at 4 to 8 weeks post-infection. The concerted action of the activated innate and adaptive immune cells successfully controls bacillary growth in rabbit lungs in the absence of significant inflammation or tissue damage. Many of the cellular activation networks were downregulated by 12 weeks, when bacterial growth declined, ultimately resulting in bacillary clearance in the absence of histopathological evidence of disease. Thus, for the first time in an animal model that spontaneously establishes LTBI similar to that in human, we show that once bacillary growth is controlled, the sustained upregulation of genes associated with activation of T cells and associated networks is dispensable to maintain latency in the host. This finding contrasts with the sustained activation of T cells and inflammation markers seen in models of chronic, active pulmonary TB in both rabbit and the mouse, [17,26].

Our transcriptomic analysis confirmed the presence of activated NK cells, and DCs in the rabbit lungs as early as 2 weeks post-CDC1551-infection. In addition, we noted upregulation of Toll-like receptors (TLR-2 and TLR-4) as early as 2 weeks that persisted until 12 weeks post-infection. Mtb-induced upregulation of TLR-2 and TLR-4 has been observed in human studies, where elevated levels of these gene transcripts were found in the blood of TB patients, compared to controls [27]. Interactions between these TLR receptors and components of the infecting bacilli facilitate activation of downstream pathways, leading to the production of proinflammatory cytokines, such as TNF-α and IL-6 [28,29]. In an acute model of Mtb respiratory infection, TLR2-/- and TLR4-/- mice showed increased susceptibility during early stages of infection, while macrophages from these mutant mice displayed reduced induction of antibacterial activities upon Mtb infection [30]. In addition, TLR-2-deficient mice showed exacerbated inflammation and succumbed to Mtb infection earlier than wild-type animals [31]. Phagocytosis of the infecting Mtb and upregulation of TLR-2 and TLR-4 would result in the early production of IL-12, thereby contributing to the activation of lymphoid cells, consistent with our observation of elevated *IL12B* expression at 2 weeks post-infection. Moreover, activated DCs and NK cells produce IFN-γ, which activates phagocytes to enhance bacterial killing [32,33]. Importantly, the expression of several key genes essential for the antimicrobial activities of macrophages, including *NOS2* and *CAP18,* were upregulated in CDC1551-infected rabbit lungs as early as 2 weeks post-infection. Inducible nitric oxide synthase (encoded by *NOS2*) is a key enzyme involved in the production of reactive nitrogen species (RNS). Phagocytosis of Mtb by activated human and murine macrophages has been shown to induce *NOS2* expression, contributing to the killing of the infecting bacilli [34-36]. Indeed, mice with a disrupted *NOS2* gene are highly susceptible to Mtb infection, showing increased lung bacillary load compared to wild-type mice, and Mtb growth is exacerbated in infected wild-type mice treated with NOS2 inhibitors compared to untreated animals [36,37]. Cathelicidin, encoded by *CAP18/CAMP*, has been shown to be involved in killing microbes, including Mtb, both in vitro and in vivo, and contributes to the host innate immune response to infection [38,39]. Upregulation of cathelicidin gene expression and stronger immunostaining for cathelicidin has been reported during Mtb infection of human alveolar macrophages, monocytes, neutrophils and epithelial cells, as well as in the lungs of infected mice [40,41]. Finally, in human and murine macrophages stimulated with cathelicidin, the extent of the LPS-mediated ROS burst was elevated [42]. These findings provide an explanation for the limited increase in the lung bacillary load during the early weeks of CDC1551 infection. The sustained activation of these effector pathways up to 8 weeks post-infection would explain how the host protective immune response contributes to bacillary containment and ultimately achieves LTBI in the rabbit lungs.

Our gene expression data indicate that peak T cell activation was achieved in the rabbit lungs by 4 weeks post-infection, consistent with our previous observations on the kinetics of T cell functionality in CDC1551-infected rabbits [15]. The expression of genes encoding cell surface markers associated with activation and differentiation of T cells, including *CD28* on CD4/8 T cells and *CD69* on DCs, as well as Th1 cytokines (*IFNG*, *IL2* and *IL15*), were maximally expressed at this time point. The co-stimulatory molecules CD28 and CD69 are essential for optimal activation of T cells and establishment of adaptive immunity [43,44]. Studies using mice conditionally expressing CD28 or treated with monoclonal antibodies to inhibit or stimulate CD28 during infection by intracellular bacteria, including Mtb, suggest that CD28 molecules are also crucial for both establishment of the initial response and the recall response in naïve and memory CD8 T cells [45]. CD69 expression was significantly elevated on CD4+ T cells obtained from the pleural fluid of TB pleurisy patients, compared to the peripheral blood of pulmonary TB patients or healthy subjects; and, upon stimulation with Mtb antigens, these cells produced increased levels of Th1 type immune molecules (IFN-γ and IL-2) [46]. Thus, the efficient recognition of Mtb antigens and successful containment of the infection were likely associated with upregulation of *CD28*, *CD69* and other genes of the T cell activation network in the CDC1551-infected rabbit lungs.

Killing of intracellular Mtb by IFN-γ activated macrophages has been shown to occur through a nitric oxide-induced apoptosis pathway [47]. In addition, increased expression of *FAS* mRNA has been noted in association with reduced intracellular growth of mycobacteria [48]. Importantly, Mtb infection of mice defective in *FAS* (CD95) failed to control bacterial growth during chronic disease [49]. Taken together, these findings are consistent with our results showing that efficient control of bacillary growth was associated with upregulation of early T cell activation network genes in the lungs of CDC1551-infected rabbits. In contrast, the limited upregulation of the B cell function network indicated that B cell activation was not prominent. These observations are supported by our previously reported functional data from CDC1551-infected rabbits [15]. Maximal antigen specific T cell proliferation in these rabbits was noted at 4 and 8 weeks for CD4+ T cells and CD8+ T cells, respectively. In contrast, activation of Mtb antigen-specific B cells was minimal, as demonstrated by the low levels of anti-PPD IgG in the circulation of the animals upon CDC1551 infection [15]. Both the molecular and functional data demonstrate that early activation of Th1 immunity is essential for the control of Mtb infection and establishment of LTBI. Interestingly, the proportion of activated T cells and the expression of the associated gene networks subsided as the bacillary load declined in the lungs.

The expression of several genes in the autophagy network was upregulated in CDC1551-infected rabbit lungs. Autophagy is a conserved cellular homeostasis process that plays a crucial role in host immunity to Mtb and other intracellular pathogens [50,51]. Only the Th1 type cytokines, such as IFN-γ, induce autophagy in macrophages to facilitate phagosomal maturation and elimination of intracellular Mtb; this process is abrogated by Th2-type cytokines, including IL-4 and IL-13 [52]. Recently, autophagy has been shown to exert antibacterial and anti-inflammatory effects against Mtb infection in a mouse model of TB, as demonstrated by the exacerbation of Mtb growth and severe inflammation in the lungs of autophagy-deficient mice compared to controls [53]. Thus, we propose that induction of autophagy is one of the mechanisms by which growth of CDC1551 is controlled in the rabbit lungs.

Genome-wide gene expression analysis is emerging as an important tool to study changes in the host immune response to infectious agents, including Mtb [54,55]. Identification and characterization of genes and networks corresponding to specific host immune responses can facilitate the identification of molecular signatures for diagnosis and monitoring of response to treatment [56-58]. Our previous reports on the Agilent rabbit microarray have relied on a partial gene annotation of rabbit genes provided by the manufacturer (Agilent Technologies Inc.) [59]. To achieve a more complete analysis of the rabbit transcriptome for this study, we have now updated the rabbit gene annotation by compiling additional rabbit genes, together with validated orthologous human and mouse genes, identified from the public genome databases. In addition, the Broad Institute is overseeing the future publication of the complete rabbit *Oryctolagus cuniculus* genome sequence. This resource will ultimately enable us to define the immunological correlates of active disease versus LTBI as well as molecular predictors of response to vaccination, drug treatment and cure of TB.

## Conclusion

Global transcriptional analysis revealed that successful control of Mtb CDC1551 infection and establishment of LTBI in rabbit lungs is associated with early upregulation of genes involved in the recruitment and activation of DCs and NK cells at 2 weeks post-infection. Thereafter, macrophage activation and the adaptive immune response networks, including antimicrobial activity and autophagy, IFN-γ function, and T cell activation are upregulated in the infected rabbit lungs, similar to the kinetics of T cell proliferation reported previously. Importantly, control of bacillary growth and establishment of latency were not associated with significant activation of inflammation. Moreover, once the bacillary load is controlled, leading to establishment of LTBI, immune activation including CD4 and CD8 T cell activation and macrophage activation are dampened. This contrasts with the host immune response to infection with Mtb HN878, which result in a sustained and larger lung bacillary load, leading to active disease driven by chronic immune activation and extensive inflammation and tissue damage. Taken together, these studies suggest that both the kinetics and the nature of the host antimicrobial response are crucial in determining the outcome following Mtb infection.

## Methods

### Bacterial culture preparation

*Mycobacterium tuberculosis* (Mtb) clinical isolate CDC1551 was provided by Dr. Shinnick (CDC, Atlanta, GA, USA). The bacterial inoculum for rabbit infection were prepared by growing Mtb to mid-log phase (OD_560_ = 0.6-0.8) in Middlebrook 7H9 medium supplemented with 10% oleic acid albumin dextrose catalase mix (Difco BD, Franklin Lakes, NJ) as described earlier [60]. Aliquots of the Mtb culture were stored at -80°C until ready to use.

### Aerosol infection of rabbits

Of the 21 female New Zealand white rabbits (*Oryctolagus cuniculus*; Millbrook Farms, Amherst, MA) used for Mtb CDC1551 aerosol infection described in a previous report, lung tissue from 18 infected animals was used for the present study [15]. In short, after a week of acclimatization to laboratory conditions, rabbits were exposed to an aerosol containing Mtb using a special “nose-only” delivery system (CH Technologies, Inc., NJ). At 3 hours post-exposure, a group of rabbits (n = 6) were euthanized and lung homogenates were placed on Middlebrook 7H10 agar medium to enumerate the initial number of implanted bacterial colony forming units (CFU). At 2, 4, 8 and 12 weeks post-infection, groups of rabbits (n = 3 per time point) were euthanized and lung tissues were harvested for CFU assay and total host RNA extraction. The detection limit for CFU assay was less than 25 CFU. A group of uninfected rabbits (n = 3) were included as controls. The procedures for rabbit infection, housing, euthanasia, necropsy and processing of Mtb containing tissues were approved by the Institutional Bio-Safety (IBC) and the Animal Care and Use Committee (IACUC) of the University of Medicine and Dentistry of New Jersey and were performed in Bio-Safety Level 3 (BSL3) containment facilities.

### RNA isolation from rabbit lungs

For total RNA isolation, portions of uninfected and Mtb-infected rabbit lung tissues, at 2, 4, 8 and 12 weeks post-infection, were snap frozen at -80°C immediately after removal.

Total host lung RNA was isolated as described [59]. Random portions of lung, representing all 5 lobes were taken for RNA isolation. In short, the snap frozen lung tissue was added to a 10× volume (wt/vol) of TRIzol reagent (Life technologies, Grand Island, NY), thawed at room temperature and homogenized on ice. The homogenate was extracted with 0.3 volumes (vol/vol) of BCP-Phase Separation Reagent (Molecular Research Center, Inc., Cincinnati, OH) and the aqueous phase was transferred to mini spin columns from the NucleoSpin RNA II kit (Macherey-Nagel GmbH&Co. Duran, Germany). After centrifugation, the column matrix containing nucleic acids was treated with DNaseI for 20 minutes at room temperature followed by purification by washing the column with buffers as described by the manufacturer. The final RNA was eluted with nuclease free water and stored at -80°C. The quantity and quality of the RNA was estimated from the OD_260_ and OD_260/280_ and OD_260/230_ values, respectively, using a NanoDrop instrument (NanoDrop products, Wilmington, DE).

### Rabbit gene expression analysis

The 4 × 44 k rabbit whole genome microarray slides, associated reagents and software were purchased from Agilent Technologies (Agilent Technologies, Inc. Santa Clara, CA) and used as per the manufacturers’ instructions. Total lung RNA from pooled, uninfected (n = 3) and three biological replicates of Mtb-infected rabbits at 2, 4, 8 and 12 weeks post-infection (n = 3 per time point) was used in the microarray experiments as described previously [59]. In short, cDNA was synthesized from one microgram of total RNA extracted from uninfected and Mtb CDC1551-infected rabbit lungs and labeled with Cy3 (uninfected) or Cy5 (Mtb-infected) respectively. Individual RNA samples from 3 animals at 2, 4, 8 and 12 weeks post-infection and pooled RNA from uninfected animals (n = 3) were used in each of the microarray experiments. The efficiency of Cy3 and Cy5 incorporation after cDNA labeling was determined by using NanoDrop instrument (NanoDrop products, Wilmington, DE). The rabbit microarray slides were hybridized with an equimolar mixture of Cy3 and Cy5 labeled cDNA. After post-hybridization washes, the slides were scanned by an Agilent Scanner and the data was acquired using Agilent Feature Extraction software (Agilent Technologies, Inc. Santa Clara, CA). The standard operating protocol for the rabbit microarray can be found at http://www.cag.icph.org. The microarray data was subjected to further statistical analysis, including Lowess error calculations using Partek Genomics Suite software version 6.5 (Partek Inc. St. Louis, MO). The statistically differentially expressed genes in the Mtb-infected animals, compared to uninfected controls, were determined from the ANOVA data by using a False Discovery Rate (FDR) of 5% (equivalent of *p* ≤ 0.05). The microarray data has been submitted to the Gene Expression Omnibus (GEO) website at http://www.ncbi.nlm.nih.gov/geo/ (accession number: GSE39219).

### Rabbit probe annotation

The commercial rabbit whole genome microarray had only 10% of 44,000 probes annotated. We increased this to 85% using ortholog mapping in five different stages. Each stage restricts its search space to those probes not annotated in the earlier stages. (1) We aligned 28,277 probes to Ensembl rabbit transcripts and those matches to the corresponding human and mouse orthologs. (2) We aligned 6361 probes to rabbit transcripts obtained from Broad Institute (unpublished data); then aligned those matches to Ensembl rabbit transcripts and eventually mapped those matches to human and mouse orthologs. (3) We mapped 1716 probes to human and mouse orthologs by first mapping them to rabbit orthologs from Broad institute and then mapping those matches to human and mouse Ensembl transcripts. (4) We also assigned human and mouse orthologs to 221 probes by directly aligning the probes to human and mouse Ensembl transcripts. (5) Finally, we imported about 300 annotations from the original Agilent annotation file that were not obtained in the previous four steps.

### Pathway analysis of rabbit lung transcriptome

The significantly differentially expressed rabbit genes (*p* ≤ 0.05) were further analyzed for functional relevance by using Ingenuity Pathway Analysis (IPA) software (Ingenuity® Systems, Inc. Redwood City, CA). Since the current IPA knowledgebase does not include the rabbit genome, we used the functional ortholog data from human, mouse and rat genomes for the pathway analysis and network derivation. In IPA, the significance of a functional pathway/network is determined by the *p*-value calculated using the right-tailed Fisher’s Exact Test. The total number of molecules in the reference set (knowledgebase) and test data set (experimental) as well as the total number of functions/pathway eligible molecules from each category are considered for the *p*-value calculations. The effect of a significantly differentially expressed transcriptional regulator is predicted by a z-score assigned by IPA, which is able to infer the activation state (increased or decreased) of a transcriptional regulator in a functional pathway/network.

### Real time quantitative PCR analysis (qPCR)

One microgram of total RNA isolated from pooled, uninfected (n = 3) and three biological replicates of Mtb-infected rabbit lungs at 2, 4, 8 and 12 weeks post-infection was used for cDNA synthesis using AffinityScript QPCR cDNA Synthesis Kit (Agilent Technologies, Inc. Santa Clara, CA). A mixture of random hexamers and oligo DT primers were used for the first strand (cDNA) synthesis. The cDNA was amplified and labeled by using gene specific oligonucleotide primers of target genes and SYBR® PremixExTaq II kit (TaKaRa Bio Inc. Shiga, Japan). The qPCR reactions were run on a Stratagene Mx 3005p machine (Agilent Technologies, Inc. Santa Clara, CA). An inert reference dye, ROX was included in all the test samples. The expression level of housekeeping *GAPDH* gene was used to normalize the expression levels of test genes in all samples. The amplicon size for all the tested genes were between 90 and 250 base pairs. Change in the level of gene expression was calculated using the formula 2^-ΔΔCt^, where ΔΔC_t_ is the difference in ΔC_t_ (threshold cycle) between the infected and uninfected samples. Description of the target genes and primer pairs used in the qPCR experiments is listed in Additional file 8: Table S6. Each experiment was repeated at least 3 times with cDNA from 3 animals at each experimental time point.

### Statistical analysis

The rabbit microarray data was analyzed by two-way ANOVA (experimental time points and infected vs. uninfected conditions) using Partek Genomics Suite software version 6.5 (Partek Inc. St. Louis, MO). Independent Student’s *t*-test from GraphPad Prism software version 5.02 (GraphPad Software, La Jolla, CA) was used for the analysis of qPCR data and the median ± standard error was plotted in graphs. For all the experiments, *p* ≤ 0.05 was considered statistically significant.

## Competing interests

The authors declare that they have no competing interests.

## Authors’ contributions

SS, LT and GK conceived the idea and designed the experiments. SS, POB, NLK, GY, LT and BP performed the experiments. NB, JSB and PCK contributed to the improved rabbit microarray gene annotation. SS, NLK, DF and GK drafted and edited the manuscript. All authors have read, understood and approved this manuscript for publication.

## Supplementary Material

Additional file 1: Table S1 Gene ontology of significantly differentially expressed rabbit genes by CDC1551 infection. The top immune cell functions perturbed in the rabbit lungs by the SDEG at 2, 4, 8 and 12 weeks post-infection. Genes were selected based on significance (FDR = 5%) and used for gene ontology analysis in IPA software. The cellular functions were ranked in a descending order (top to bottom) based on the total number of perturbed genes at 2 weeks post-infection. Click here for file

Additional file 2: Table S2 Canonical pathways affected by the SDEG in the CDC1551-infected rabbit lungs. The top canonical pathways perturbed in the rabbit lungs by the SDEG at 2, 4, 8 and 12 weeks post-infection. Genes were selected based on significance (FDR = 5%) and used for canonical pathway analysis in IPA software. The pathways were ranked in a descending order (top to bottom) based on the *p*-value (-log scale) at 2 weeks post-infection. Click here for file

Additional file 3: Table S3 Cellular functions affected by SDEGs in the CDC1551-infected rabbit lungs. The top cellular functions affected by the SDEG in the rabbit lungs at 2, 4, 8 and 12 weeks post-infection. The functions were predicted by the z-value calculations based on the effect of downstream transcriptional regulators (TR) in IPA software. A positive z-score indicates activation and negative z-score means inhibition of a respective cellular function. Only z-values greater than +2 or less than -2 were considered significant. Also shown is total number of SDEG and the *p*-value that shows the cellular function affected significantly only by CDC1551 infection and not a random effect. The cellular functions were ranked in an ascending fashion (top to bottom) based on the z-score at each time point. Click here for file

Additional file 4: Table S4 Significantly differentially expressed transcriptional regulators and their impact on the cell function in the CDC1551-infected rabbit lungs. List of top TR among the SDEG in the rabbit lungs at 2, 4, 8 and 12 weeks post-infection and their effect on cellular function. The functions were predicted by the z-value calculations based on the expression pattern of the downstream TR in IPA software. A positive z-score indicates activation and negative z-score means inhibition of a respective cellular function. Only z-values greater than +2 or less than -2 were considered significant. Also shown is total number of SDEG regulated by a TR and the *p*-value that shows the cellular function affected significantly only by CDC1551 infection and not a random effect. The cellular functions were ranked in an ascending fashion (top to bottom) based on the z-score at each time point. Click here for file

Additional file 5: Table S5 Validation of microarray gene expression results by quantitative real time PCR (qPCR). Seventeen genes were randomly chosen from the microarray data (MA) corresponding to 2, 4, 8 and 12 weeks post-infection and their expression levels were measured by qPCR method. The qPCR values shown are median and standard error (se) from 3 biological replicate samples at each time point. Expression level of each gene in every sample was calibrated against house-keeping, GAPDH levels. Positive values indicate up-regulation and negative value represents down-regulation. Click here for file

Additional file 6: Figure S1 Canonical pathway for the communication between DCs and NK cells. The significantly differentially expressed rabbit genes at 2 weeks post-infection was used to derive the canonical pathway map in IPA software. Red colored symbols in the pathway indicates up-regulation and green color denotes down-regulation of gene expression and the gradation in the color intensity is proportional to their relative expression level. No color indicates absence or insignificant level of expression. The legend is same as in Figure 2. Click here for file

Additional file 7: Figure S2 Canonical pathway for the interaction between cells of the innate and adaptive immune response. The SDEG at 4 weeks post-infection were used to construct the canonical pathway map in IPA software. Red symbols in the pathway indicate up-regulation; green denotes down-regulation of gene expression; and the gradation in color intensity is proportional to relative expression levels. Colorless indicates absence or insignificant level of expression. The legend is same as in Figure 2. Click here for file

Additional file 8: Table S6 Description of oligonucleotide primers used in the quantitative real time PCR (qPCR) experiments. Gene sequence for primer design was obtained from public data base using the gene IDs provided in column 5. The amplicon size for the all the genes mentioned in the list were between 90 and 250 base pairs. Click here for file
