# Supplementary figures and images for: Molecular immunologic correlates of spontaneous latency in a rabbit model of pulmonary tuberculosis
Source: Cell Commun Signal. 2013 Feb 28;11:16. doi: 10.1186/1478-811X-11-16 (PMC3598925; doi:10.1186/1478-811X-11-16)

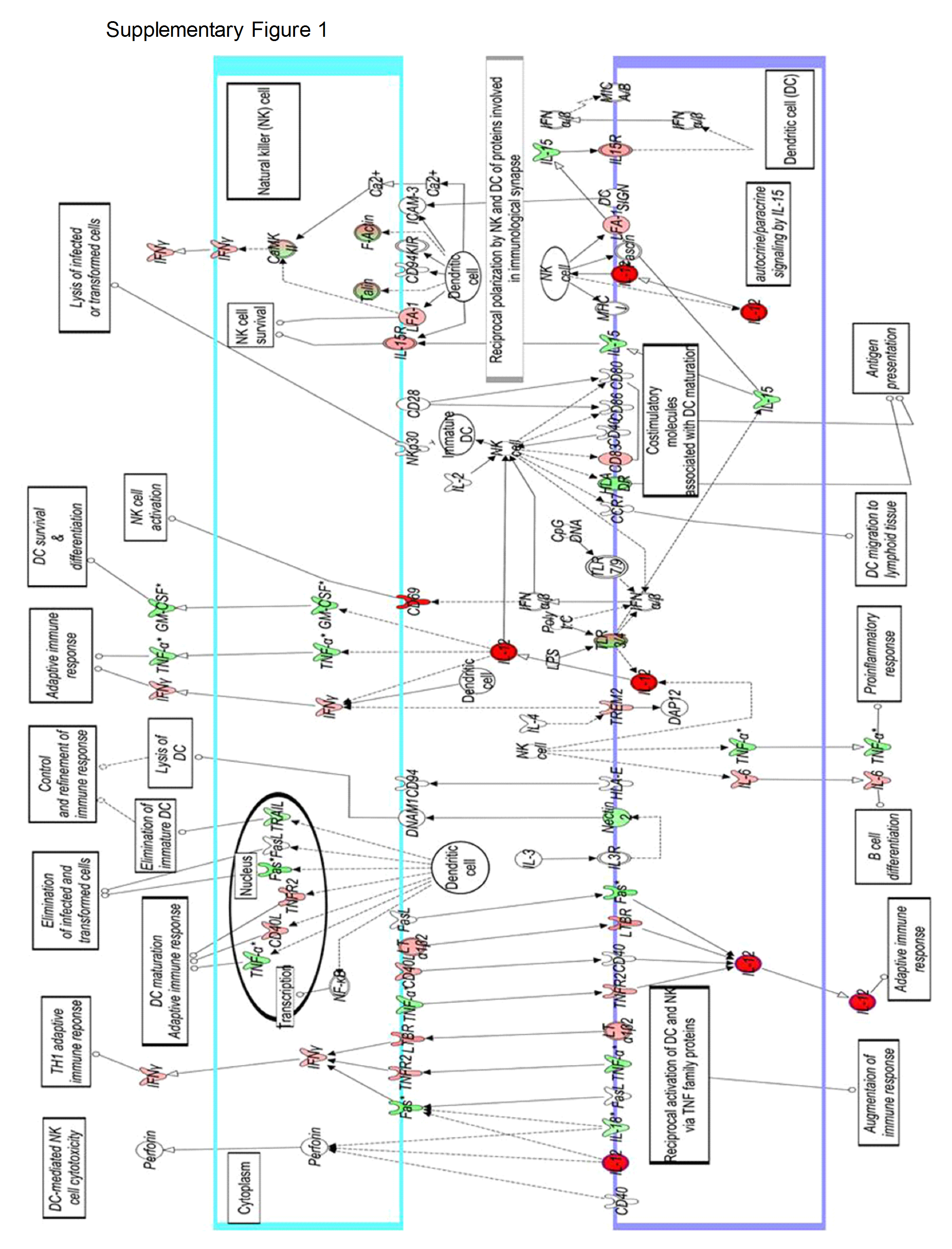

Supplement: Additional file 6: Figure S1 — Canonical pathway for the communication between DCs and NK cells. The significantly differentially expressed rabbit genes at 2 weeks post-infection was used to derive the canonical pathway map in IPA software. Red colored symbols in the pathway indicates up-regulation and green color denotes down-regulation of gene expression and the gradation in the color intensity is proportional to their relative expression level. No color indicates absence or insignificant level of expression. The legend is same as in Figure 2. [file 1478-811X-11-16-S6.tiff]

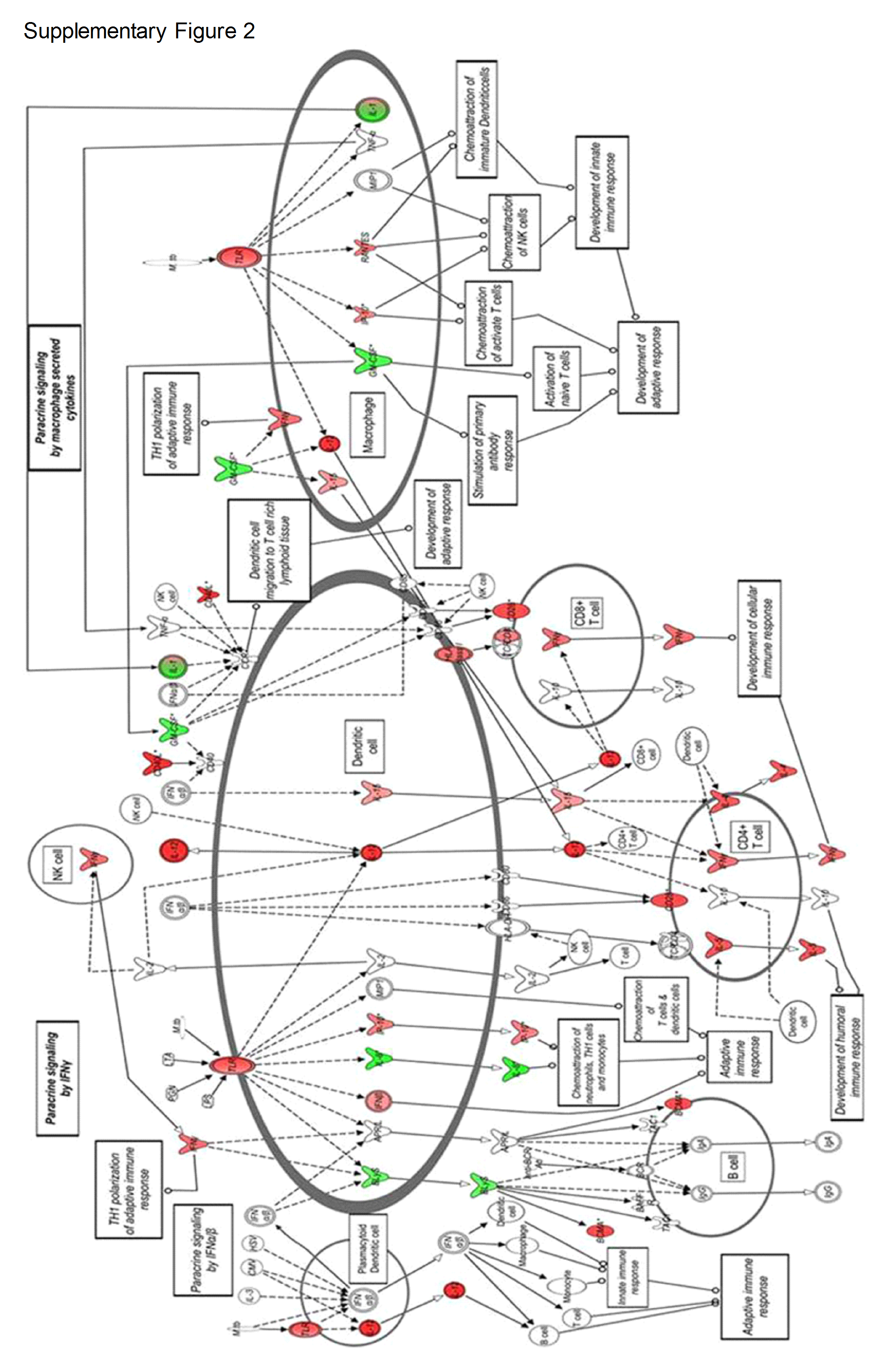

Supplement: Additional file 7: Figure S2 — Canonical pathway for the interaction between cells of the innate and adaptive immune response. The SDEG at 4 weeks post-infection were used to construct the canonical pathway map in IPA software. Red symbols in the pathway indicate up-regulation; green denotes down-regulation of gene expression; and the gradation in color intensity is proportional to relative expression levels. Colorless indicates absence or insignificant level of expression. The legend is same as in Figure 2. [file 1478-811X-11-16-S7.tiff]
